# Laboratory-Negative Results as a Marker of Diagnostic Gap: A Retrospective Study in the One Health Context

**DOI:** 10.7759/cureus.108198

**Published:** 2026-05-03

**Authors:** Prachee Singh

**Affiliations:** 1 Department of Microbiology, Ajay Sangaal Institute of Medical Sciences and Research, Shamli, IND

**Keywords:** antimicrobial stewardship, diagnostic gap, diagnostic stewardship, laboratory-negative results, one health

## Abstract

Background: Antimicrobial resistance (AMR) is a major global health concern, driven in part by inappropriate antimicrobial use and diagnostic uncertainty. Laboratory-based diagnostics play a critical role in guiding rational therapy; however, a high proportion of laboratory-negative results may reflect potential gaps in diagnostic processes.

Objective: The objective of this study is to assess the proportion of laboratory-confirmed infections among patients undergoing microbiological investigations and to explore a potential diagnostic gap using laboratory-negative results as an indirect and exploratory indicator.

Methods: This retrospective cross-sectional study was conducted in the Department of Microbiology of a tertiary care teaching hospital. A total of 1,304 microbiological samples, including serological, bacteriological, and mycological investigations, were analyzed. Data were extracted from laboratory records, and variables such as age, sex, department (OPD/IPD), type of investigation, and laboratory results were assessed. Positivity rates were compared across diagnostic modalities. Statistical analysis was performed using the chi-square test, with a p-value of <0.05 considered statistically significant.

Results: Serological investigations constituted the majority of samples (86.4%). The overall laboratory positivity rate was 19.9%, with 80.1% of samples showing no confirmed infection. Bacteriological investigations demonstrated a higher positivity rate (59.2%) compared to serology and mycology. Positivity rates varied across age groups, with higher rates observed among elderly patients. Modality-specific differences in diagnostic yield were observed, suggesting variability in test utilization patterns.

Conclusion: This study demonstrates a high proportion of laboratory-negative results, indicating a potential diagnostic gap in clinical practice. These findings should be interpreted cautiously due to the influence of pre-analytical factors, analytical limitations, and variations in test utilization. Strengthening diagnostic stewardship and promoting microbiology-guided therapy are essential to optimize antimicrobial use. The results are interpreted within a one health perspective as a conceptual framework. Further prospective studies incorporating clinical correlation and antimicrobial exposure are required.

## Introduction

Antimicrobial resistance (AMR) is increasingly recognized as a critical global health challenge that threatens the effectiveness of currently available antimicrobial agents. The increasing prevalence of resistant microorganisms compromises the effectiveness of commonly used antimicrobial agents, resulting in prolonged illness, increased mortality, and rising healthcare costs. Low- and middle-income countries such as India bear a disproportionately high burden of AMR due to the high prevalence of infectious diseases, widespread antibiotic use, and limited diagnostic infrastructure [1].

In routine clinical practice, antibiotic treatment is often initiated empirically before confirmation of the causative organism. This approach is often necessary in situations requiring rapid clinical decision-making, particularly in suspected severe infections where culture and sensitivity results may take 24-72 hours. However, excessive reliance on empirical therapy without adequate microbiological evidence contributes to inappropriate antibiotic use and accelerates the emergence and spread of AMR [2].

India ranks among the highest antibiotic-consuming countries worldwide, with a marked increase in usage over recent decades [3]. Inappropriate prescribing practices, self-medication, and over-the-counter availability of antibiotics further contribute to irrational antimicrobial use in both community and hospital settings [4]. These factors have significantly contributed to the rising burden of AMR among clinically important pathogens in the country.

Laboratory-based surveillance systems play a critical role in monitoring AMR and guiding rational antibiotic therapy. Microbiological testing is essential for identifying causative pathogens and determining their antimicrobial susceptibility patterns, thereby supporting evidence-based treatment decisions. The establishment of AMR surveillance networks in India has strengthened the availability of resistance data and facilitated antimicrobial stewardship initiatives [5].

Recognizing the growing threat of AMR, India has implemented the National Action Plan on Antimicrobial Resistance (NAP-AMR), which emphasizes surveillance, antimicrobial stewardship, infection prevention and control, and the adoption of the One Health approach that integrates human, animal, and environmental health sectors [6]. Water systems serve as multifaceted environmental pools for antibiotic-resistant bacteria and resistance genes, affecting human, animal, and environmental health [7].

The One Health approach, promoted by the World Health Organization, is based on the recognition that optimal human health cannot be achieved by focusing solely on human healthcare. Instead, it requires coordinated efforts across multiple sectors, including veterinary sciences, agriculture, and environmental sciences. The use of antibiotics in animals, particularly for growth promotion or prophylaxis, and their subsequent entry into the human food chain may contribute cumulatively to the development of AMR in humans. Similarly, antimicrobial use in agriculture and environmental reservoirs further contributes to this interconnected risk. In line with WHO recommendations, governments have established intersectoral coordination mechanisms involving stakeholders from human health, animal health, agriculture, and environmental sectors to implement the One Health strategy effectively at the ground level.

Despite these efforts, there remains a significant gap in integrated research. Among 2,152 AMR-related studies from India, 48.3% focused on human health, while only 3.3% addressed animal health, 4.2% the environment, and only 0.5% specifically focused on the One Health domain, highlighting the need for more multidisciplinary research.

Antimicrobial stewardship programs are essential for optimizing antibiotic use, improving patient outcomes, and minimizing unnecessary antimicrobial exposure. Evidence suggests that such interventions significantly improve antimicrobial utilization in healthcare settings [8]. However, empirical antibiotic prescribing remains common due to diagnostic uncertainty and delays in microbiological confirmation.

Studies on acute undifferentiated febrile illness in India have shown that only a subset of patients receive a confirmed cause despite extensive testing [9]. Additionally, prior antibiotic use has been found to significantly lower blood culture positivity rates; for instance, among patients with sepsis, positivity was 50.6% in those not on antibiotics compared to 27.7% in those already undergoing treatment [10,11].

The absence of microbiological confirmation contributes to a potential diagnostic gap between empirical treatment and evidence-based therapy. Antimicrobial exposure before specimen collection may reduce pathogen detection and contribute to laboratory-negative findings; however, this relationship was not directly assessed in the present study.

In the context of this study, the term “diagnostic gap” refers to the discrepancy between clinical suspicion of infection requiring microbiological investigation and the absence of laboratory-confirmed evidence of infection. This gap is explored using laboratory-negative results as an indirect and exploratory indicator, recognizing that it does not represent a direct measurement.

In this context, systematic evaluation of microbiological investigation patterns can provide important insights into diagnostic practices and laboratory-confirmed infections. However, there is limited evidence from Indian tertiary care settings examining this diagnostic gap across multiple microbiological domains (serology, bacteriology, and mycology).

Therefore, the present retrospective laboratory-based observational study was conducted to analyze patterns of microbiological investigations and laboratory-confirmed infections in a tertiary care hospital. By identifying the proportion of laboratory-negative results as an indirect indicator of a potential diagnostic gap, the study aims to highlight gaps in diagnostic stewardship. The findings are interpreted within a One Health perspective, recognizing the interconnected role of human, animal, and environmental factors in AMR. This perspective is applied as a conceptual framework rather than an operational component of the study design.

## Materials and methods

Aim/objective of the study

The study aims to assess the proportion of laboratory-confirmed infections among patients undergoing microbiological investigations and to explore a potential diagnostic gap using laboratory-negative results as an indirect and exploratory indicator in a tertiary care hospital (Ajay Sangaal Institute of Medical Sciences and Research, Shamli). The study was conducted within a human healthcare setting, and the findings are interpreted within a One Health perspective as a conceptual framework rather than an operational component of the study design.

Study design and setting

This retrospective cross-sectional observational study was conducted in the Department of Microbiology at a tertiary care teaching hospital, serving both inpatient and outpatient populations.

Study duration

The study was carried out over a period of three months.

Study population and sampling technique

All microbiological samples received in the laboratory during the study period were included using a consecutive sampling technique. These comprised serological, bacteriological, and mycological investigations from both outpatient (OPD) and inpatient (IPD) departments.

Sample size

A total of 1,304 patient samples were analyzed during the study period.

Inclusion criteria

Inclusion criteria were as follows: (i) all microbiological samples received during the study period; (ii) samples from both OPD and IPD patients; and (iii) samples processed for serological, bacteriological, or mycological investigations.

Exclusion criteria

Exclusion criteria were as follows: (i) repeat samples from the same patient; (ii) samples with incomplete records or missing data; and (iii) samples with indeterminate or borderline diagnostic results.

Data extraction process

Data were extracted retrospectively from laboratory records and registers using a standardized data collection format. Extracted variables included patient demographics (age and sex), type of investigation (serology, bacteriology, and mycology), department (OPD/IPD), and laboratory results. Data were anonymized prior to analysis, and records with incomplete or missing key variables were excluded.

Definition of laboratory results

A result was considered laboratory positive when a clinically relevant pathogen was identified by culture or when serological testing yielded a reactive/positive result based on predefined kit-specific criteria. Results were considered laboratory-negative when no pathogen was isolated in culture or when serological tests were non-reactive.

Laboratory procedures

All samples were processed according to the standard operating procedures of the microbiology laboratory.

Specimen Types

The study included a range of clinical specimens such as blood, urine, respiratory samples (sputum), pus/wound swabs, body fluids, and serum samples, depending on the type of investigation requested.

Bacteriological Investigations

Samples were processed using standard culture techniques on appropriate media such as blood agar, MacConkey agar, and other selective media as indicated. Identification of isolates was performed using conventional biochemical methods as per standard microbiological protocols.

Mycological Investigations

Fungal samples were processed using direct microscopy (e.g., KOH mount) and cultured on appropriate fungal media such as Sabouraud dextrose agar, followed by standard identification methods.

Serological Investigations

Serological tests were performed using commercially available diagnostic kits (e.g., ELISA and rapid immunochromatographic assays) according to manufacturer instructions. Results were interpreted as positive/reactive or negative/non-reactive based on predefined kit-specific cut-off values.

Routine internal quality control procedures were followed for all investigations.

Variables and outcome measures

The variables analyzed included type of investigation (serology, bacteriology, mycology), age, sex, department (OPD/IPD), and laboratory positivity rates.

The primary outcome measure was the proportion of laboratory-confirmed infections. Laboratory-negative results were analyzed as an indirect (proxy) and exploratory indicator to assess a potential diagnostic gap. In this study, the diagnostic gap refers to the discrepancy between clinical suspicion of infection requiring microbiological investigation and the absence of laboratory-confirmed evidence of infection.

Pre-analytical variables and confounding factors

Pre-analytical factors such as prior antibiotic exposure, timing and quality of specimen collection, sample handling, and transport conditions may influence microbiological yield. Clinical factors such as severity of illness and indications for testing may also affect laboratory results.

These variables were not available in the retrospective dataset and were therefore not directly analyzed; however, their potential impact has been considered in the interpretation of findings.

Antibiotic Exposure

Information regarding antimicrobial administration prior to or after specimen collection was not available in the laboratory records and could not be assessed in this study.

Statistical analysis

Data were analyzed using IBM SPSS Statistics for Windows, Version 25 (Released 2017; IBM Corp., Armonk, New York, United States). Descriptive statistics were used to summarize categorical variables as frequencies and percentages. The chi-square test was applied to compare proportions between groups. A p-value of <0.05 was considered statistically significant.

Ethical considerations

This study was conducted using anonymized retrospective laboratory data derived from routine clinical samples. No direct patient interaction or intervention was involved. Institutional Ethics Committee approval was waived as per institutional policy. Informed patient consent was waived, and no identifiable patient information is included in this study.

## Results

A total of 1,304 microbiological samples were analyzed during the study period. Serological investigations constituted the majority of samples (1,127; 86.4%), followed by bacteriological (130; 10.0%) and mycological investigations (47; 3.6%) (Table 1).

**Table 1:** Distribution of Samples by Speciality

Specialty	Total Samples Tested	Positive n (%)	Negative n (%)
Serology	1127	170 (15.1%)	957 (84.9%)
Bacteriology	130	77 (59.2%)	53 (40.8%)
Mycology	47	13 (27.7%)	34 (72.3%)
Total	1304	260 (19.9%)	1044 (80.1%)

The overall laboratory positivity rate was 19.9% (260/1304), while 80.1% (1044/1304) of samples showed no confirmed infection (Table 1).

Among different diagnostic modalities, bacteriological samples demonstrated the highest positivity rate (59.2%), followed by mycological (27.7%) and serological investigations (15.1%) (Table 1).

Sex-wise distribution showed that female patients constituted a higher proportion of samples (57.2%) compared to male patients (42.8%). However, positivity rates were comparable across sexes, and no statistically significant difference was observed (Table 2).

**Table 2:** Sex-Wise Distribution

Specialty	Total Number of Male Patients Tested n (%)	Male Positive n (%)	Total Number of Female Patients Tested n (%)	Female Positive n (%)
Serology	460 (40.8%)	69 (15.0%)	667 (59.2%)	101 (15.1%)
Bacteriology	71 (54.6%)	41 (57.7%)	59 (45.4%)	36 (61.0%)
Mycology	27 (57.4%)	8 (29.6%)	20 (42.6%)	5 (25.0%)
Total	558 (42.8%)	118 (21.1%)	746 (57.2%)	142 (19.0%)

Age-wise analysis revealed that patients aged >60 years constituted the largest proportion of samples and demonstrated higher positivity rates across all categories. In serological investigations, the positivity rate in this age group was 26.8%, while bacteriological and mycological investigations showed positivity rates of 67.9% and 34.8%, respectively (Tables 3-5).

**Table 3:** Age-Wise Distribution – Serology

Age Group (Years)	Total Tested n (%)	Positive n (%)	Negative n (%)
<10	19 (1.7%)	1 (5.3%)	18 (94.7%)
11–20	66 (5.9%)	2 (3.0%)	64 (97.0%)
21–30	167 (14.8%)	10 (6.0%)	157 (94.0%)
31–40	160 (14.2%)	12 (7.5%)	148 (92.5%)
41–50	195 (17.3%)	28 (14.4%)	167 (85.6%)
51–60	203 (18.0%)	32 (15.8%)	171 (84.2%)
≥60	317 (28.1%)	85 (26.8%)	232 (73.2%)
Total	1127 (100.0%)	170 (15.1%)	957 (84.9%)

**Table 4:** Age-Wise Distribution – Bacteriology

Age Group (Years)	Total Tested n (%)	Positive n (%)	Negative n (%)
<10	2 (1.5%)	2 (100.0%)	0 (0.0%)
11–20	6 (4.6%)	3 (50.0%)	3 (50.0%)
21–30	6 (4.6%)	2 (33.3%)	4 (66.7%)
31–40	11 (8.5%)	5 (45.5%)	6 (54.5%)
41–50	24 (18.5%)	13 (54.2%)	11 (45.8%)
51–60	28 (21.5%)	16 (57.1%)	12 (42.9%)
≥60	53 (40.8%)	36 (67.9%)	17 (32.1%)
Total	130 (100.0%)	77 (59.2%)	53 (40.8%)

**Table 5:** Age-Wise Distribution – Mycology

Age Group (Years)	Total Tested n (%)	Positive n (%)	Negative n (%)
<10	0 (0.0%)	0 (0.0%)	0 (0.0%)
11–20	1 (2.1%)	1 (100.0%)	0 (0.0%)
21–30	1 (2.1%)	0 (0.0%)	1 (100.0%)
31–40	2 (4.3%)	0 (0.0%)	2 (100.0%)
41–50	6 (12.8%)	1 (16.7%)	5 (83.3%)
51–60	14 (29.8%)	3 (21.4%)	11 (78.6%)
≥60	23 (48.9%)	8 (34.8%)	15 (65.2%)
Total	47 (100.0%)	13 (27.7%)	34 (72.3%)

Department-wise distribution indicated that the majority of samples were received from inpatient departments (IPD: 85.5%) compared to outpatient departments (OPD: 14.5%). The proportion of negative results was high in both IPD (80.1%) and OPD (79.9%) settings (Table 6).

**Table 6:** Department-Wise Distribution

Specialty	OPD Tested n (%)	OPD Positive n (%)	OPD Negative n (%)	IPD Tested n (%)	IPD Positive n (%)	IPD Negative n (%)	Total
Medicine	98 (7.5%)	19 (19.4%)	79 (80.6%)	627 (48.1%)	127 (20.3%)	500 (79.7%)	725
Orthopedics	12 (0.9%)	3 (25.0%)	9 (75.0%)	55 (4.2%)	10 (18.2%)	45 (81.8%)	67
Gynecology	12 (0.9%)	3 (25.0%)	9 (75.0%)	150 (11.5%)	29 (19.3%)	121 (80.7%)	162
Surgery	24 (1.8%)	5 (20.8%)	19 (79.2%)	198 (15.2%)	39 (19.7%)	159 (80.3%)	222
Others	43 (3.3%)	8 (18.6%)	35 (81.4%)	85 (6.5%)	17 (20.0%)	68 (80.0%)	128
Total	189 (14.5%)	38 (20.1%)	151 (79.9%)	1115 (85.5%)	222 (19.9%)	893 (80.1%)	1304

Overall, across all microbiological investigations, a consistently high proportion of laboratory-negative results (80.1%) was observed.

## Discussion

AMR remains a major global health concern, driven in part by inappropriate antibiotic use. Addressing this challenge requires strengthening microbiological diagnostics and promoting rational antimicrobial utilization.

The present study evaluated microbiological investigation patterns in a tertiary care setting and identified a predominance of serological testing (86.4%), reflecting its widespread use due to rapid turnaround time and ease of performance. Hospital-based studies have reported similar trends, with serology serving as a primary screening tool for infectious diseases [12,13].

A notable finding in this study is the predominance of serological investigations, which constituted the majority of samples analyzed. This skewed distribution may influence the overall positivity rate, as different diagnostic modalities have inherently varying yields. Therefore, overall positivity estimates should be interpreted with caution, and greater emphasis should be placed on modality-specific positivity rates. The higher positivity observed in bacteriological investigations compared to serological tests further supports this interpretation and highlights differences in test utilization patterns [12,13].

The overall positivity rate of 19.9% indicates that a large proportion of samples did not yield confirmed infectious etiologies. Similar findings have been reported in Indian hospital settings, where positivity rates vary according to specimen type and clinical context [14,15].

Bacteriological samples showed a significantly higher positivity rate (59.2%) compared to serology and mycology. This may reflect more targeted test utilization based on stronger clinical suspicion, whereas lower positivity in other modalities may indicate broader or less selective test ordering practices, differences in diagnostic performance, or underlying disease etiology [14,15]. These modality-specific differences provide important context for interpreting laboratory-negative results. Previous studies have similarly emphasized the role of microbiological culture and susceptibility testing in antimicrobial stewardship [16,17].

An analysis by sex revealed a greater percentage of female patients, although positivity rates were comparable between genders. This suggests that differences in sample distribution may reflect healthcare-seeking behavior rather than variation in infection susceptibility, consistent with earlier studies [18,19].

Age-wise findings revealed that elderly patients (>60 years) constituted the largest proportion of cases and demonstrated higher positivity rates across all categories. Immunosenescence, comorbidities, and greater healthcare exposure may contribute to increased susceptibility in this group [20,21]. Prolonged hospitalization, prior antibiotic exposure, and underlying illnesses may also contribute to the higher burden of fungal infections in older individuals [22,23].

A key finding of this study is that 80.1% of samples were negative for confirmed infection. While this may suggest a potential diagnostic gap, this observation should be interpreted cautiously. Laboratory-negative results may arise from multiple factors, including pre-analytical variables such as prior antibiotic exposure, timing and quality of specimen collection, analytical limitations of conventional diagnostic methods, and variations in test utilization patterns.

Although empirical antimicrobial therapy may be initiated prior to laboratory confirmation in clinical practice, this relationship was not directly assessed in the present study. Therefore, the findings should be considered exploratory and hypothesis-generating rather than confirmatory [24,25].

The interpretation of a potential diagnostic gap in this study is supported by both the overall proportion of laboratory-negative results and the variation in positivity rates across different diagnostic modalities. However, these findings should be interpreted cautiously, as the diagnostic gap is inferred as an indirect and exploratory concept rather than a direct measurement.

Laboratory-negative results may arise from multiple factors. Pre-analytical factors such as prior antibiotic exposure, timing of specimen collection, and sample quality can significantly influence microbiological yield. Analytical limitations, including the sensitivity constraints of conventional diagnostic methods, may also result in false-negative findings. Etiological factors such as viral or non-infectious conditions may account for negative results despite clinical suspicion of infection.

In addition, variations in test utilization patterns, including non-targeted or routine ordering of investigations, particularly serological tests, may contribute to lower diagnostic yield. The predominance of serological investigations in this study, coupled with their comparatively lower positivity rates, raises the possibility of broader or less selective test utilization in routine clinical practice.

While serological tests offer advantages such as rapid turnaround time and ease of use, their overutilization without appropriate clinical indication may reduce diagnostic yield and delay identification of definitive etiological agents. This has important implications for antimicrobial stewardship, as suboptimal diagnostic strategies may lead to continued empirical therapy in the absence of confirmatory evidence.

These findings emphasize the need for strengthening diagnostic stewardship, including more targeted and clinically guided test selection, improved clinician-laboratory coordination, and timely specimen collection before initiation of antimicrobial therapy.

Department-wise analysis indicated that most samples were from IPD patients, with comparable positivity rates between OPD and IPD settings. However, the higher volume of negative reports in hospitalized patients may reflect multiple contributing factors, including severity of illness, early empirical therapy, and diagnostic practices.

From a One Health perspective, AMR is a complex issue involving interconnected human, animal, and environmental systems. Although this study is grounded in a human healthcare setting, the findings are interpreted within a One Health perspective to highlight the broader implications of AMR. It is important to note that the One Health approach is not operationalized in the study design, as data from animal and environmental domains were not included. Therefore, it is used here as a conceptual framework rather than a directly applied methodological approach [26,27].

Overall, this study provides real-world insights into microbiological investigation patterns and highlights the importance of optimizing diagnostic practices. The findings should be interpreted as exploratory and hypothesis-generating, providing a basis for future prospective studies incorporating clinical correlation, antimicrobial exposure, and diagnostic decision-making patterns.

Limitations

This study has several limitations. It is a retrospective, single-center analysis, which may limit generalizability. Data on prior antibiotic exposure, timing, and quality of specimen collection, and clinical indications for testing were not available, preventing direct assessment of their impact on microbiological outcomes. Clinical correlation with patient outcomes was also not evaluated. Additionally, variations in diagnostic test utilization and the predominance of certain investigation types may have influenced overall positivity rates. In particular, the skewed distribution of samples, with a predominance of serological investigations (86.4%), may have affected the overall proportion of laboratory-positive and laboratory-negative results. Therefore, overall positivity rates should be interpreted with caution, with greater emphasis on modality-specific findings. A key interpretative limitation is that laboratory-negative results were used as an indirect (proxy) indicator to explore a potential diagnostic gap. This approach does not provide direct evidence and should be interpreted cautiously. Additionally, the study does not operationalize the One Health framework, as it is limited to human healthcare data without the inclusion of animal or environmental components.

## Conclusions

This study demonstrates a high proportion of laboratory-negative results across microbiological investigations, which indicates a potential diagnostic gap in routine clinical practice. However, this observation should be interpreted cautiously, as laboratory-negative results may be influenced by multiple factors, including pre-analytical variables, analytical limitations, and variations in test utilization, which were not directly assessed in this study.

The findings are exploratory and highlight the importance of strengthening diagnostic stewardship through targeted test utilization, timely specimen collection, and microbiology-guided therapy to optimize antimicrobial use. Although based on human healthcare data, the results are interpreted within a One Health perspective as a conceptual framework. Further prospective studies incorporating clinical correlation and antimicrobial exposure are warranted.
